# Contrasting environmental drivers of adult and juvenile growth in a marine fish: implications for the effects of climate change

**DOI:** 10.1038/srep10859

**Published:** 2015-06-08

**Authors:** Joyce Jia Lin Ong, Adam Nicholas Rountrey, Jessica Jane Meeuwig, Stephen John Newman, Jens Zinke, Mark Gregory Meekan

**Affiliations:** 1 School of Animal Biology and the Centre for Marine Futures (UWA Oceans Institute M096), University of Western Australia, 35 Stirling Highway, Crawley, Western Australia 6009; 2 Australian Institute of Marine Science, UWA Oceans Institute (M096), University of Western Australia, 35 Stirling Highway, Crawley, Western Australia 6009; 3 Museum of Paleontology, University of Michigan, 1109 Geddes Avenue, Ann Arbor, Michigan 48109-1079, United States of America; 4 Western Australian Fisheries and Marine Research Laboratories, Department of Fisheries, Government of Western Australia, PO Box 20, North Beach, Western Australia 6920; 5 School of Earth and Environment and the UWA Oceans Institute (M096), University of Western Australia, 35 Stirling Highway, Crawley, Western Australia 6009; 6 School of Geography, Archaeology and Environmental Studies, University of Witwatersrand, Johannesburg, South Africa; 7 Curtin University of Technology, Department of Environment and Agriculture, Kent Street, Perth, Western Australia 6845

## Abstract

Many marine fishes have life history strategies that involve ontogenetic changes in the use of coastal habitats. Such ontogenetic shifts may place these species at particular risk from climate change, because the successive environments they inhabit can differ in the type, frequency and severity of changes related to global warming. We used a dendrochronology approach to examine the physical and biological drivers of growth of adult and juvenile mangrove jack (*Lutjanus argentimaculatus*) from tropical north-western Australia. Juveniles of this species inhabit estuarine environments and adults reside on coastal reefs. The Niño-4 index, a measure of the status of the El Niño-Southern Oscillation (ENSO) had the highest correlation with adult growth chronologies, with La Niña years (characterised by warmer temperatures and lower salinities) having positive impacts on growth. Atmospheric and oceanographic phenomena operating at ocean-basin scales seem to be important correlates of the processes driving growth in local coastal habitats. Conversely, terrestrial factors influencing precipitation and river runoff were positively correlated with the growth of juveniles in estuaries. Our results show that the impacts of climate change on these two life history stages are likely to be different, with implications for resilience and management of populations.

Many fishes of high commercial value have life history strategies in which successive ontogenetic stages occupy different habitats. Change in habitat can be relatively minor, such as the transition from inshore to deeper offshore waters in Atlantic herring (*Clupea harengus*) and cod (*Gadus morhua*)^1^,^2^, or more extreme as observed in European seabass (*Dicentrarchus labrax*) and giant trevally (*Caranx ignobilis*), where juveniles are found in estuarine nurseries, while adults are found in fully marine habitats offshore^3^. Perhaps the most extreme examples of habitat change between ontogenetic stages are shown by species that are catadromous (e.g. barramundi, *Lates calcarifer*) and anadromous (e.g. chinook salmon, *Oncorhynchus tshawytscha*). In the case of the former, adults are found in brackish or freshwater habitats while larvae and juveniles occupy marine habitats. In the latter, larvae and juveniles are found in freshwater and the adults reside offshore^3^.

Species that undergo ontogenetic shifts of habitat are thought to do so to avoid predators and/or to improve foraging, so that there is a selective balance between minimizing mortality risk and maximising energy gains^4^. However, marine habitats are changing due to anthropogenic processes such as global warming and ocean acidification. The need to occupy a range of habitats during each life history stage could make such species particularly vulnerable to increased climate variability, because impacts could differ in type, frequency and severity in any of the successive habitats they occupy. For example, sea-level rise and associated intrusions of salt water could reduce the area of estuarine nursery habitats^5^ used by juveniles of many species. Estuarine waters are also expected to undergo accelerated warming because of their small size and shallow depths^6^. Additionally, changes in rainfall and terrestrial run-off patterns may alter freshwater inputs, changing salinities and the stratification of the water column^6^. These in turn could lead to reduced water quality and hyper-saline conditions. In coastal waters, sea surface temperatures are rising and patterns of current flow are changing. Although warmer sea surface temperatures are likely to increase the growth rates of fishes living at sub-optimal temperatures^7^, for individuals and species living closer to thermal optima, warmer waters might decrease growth rates^8^ and cause range shifts and contractions^9^.

At present, what we know of the likely response to climate change by many marine fishes tends to be based on observations of the adult life history stage^10^. Given that the consequences of climate change may vary among habitats, obligate ontogenetic shifts in habitat may make some species more vulnerable than others that are largely sedentary, complicating predictions of species responses. For this reason, studies are required that assess the key physical and environmental drivers of growth at different life history stages of marine fishes that undergo ontogenetic habitat shifts.

This issue is of particular concern in the tropics where fish assemblages in estuarine and shelf waters provide the major source of protein for more than 600 million people in Asia^11^ and more than 200 million people in Africa^12^, most of whom live in developing countries. There are numerous examples of tropical species that spawn at sea and enter estuaries as juveniles, including mangrove jack (*Lutjanus argentimaculatus*), giant trevally (*C. ignobilis*), milkfish (*Chanos chanos*) and Genus of this species has changed from Liza to Chelon. (*Chelon macrolepis*)^3^. Such species are central to tropical fisheries, largely because major population centres tend to be clustered on the coasts that surround estuarine habitats.

Here, we examine the impacts of past environmental variation on different life history stages of the mangrove jack as a means of assessing likely consequences of future climate change. This species has a juvenile stage that occurs in mangroves, estuaries and some freshwater habitats that are heavily influenced by ephemeral processes such as river runoff and rainfall. In contrast, adults are found on tropical coastal and offshore reefs, a more stable environment affected by oceanic factors. The physical and biological processes within these different environments are expected to affect individual fish via both physiological and ecological mechanisms that in turn, affect their growth. Hence, we hypothesize that, in the warmer months of January to March (the summer growing season), juvenile growth is likely to respond to factors that affect the productivity of estuarine systems such as rainfall, whereas adult growth will respond to factors that affect the productivity of coastal ecosystems such as Sea Surface Temperature (SST) and Sea Surface Salinity (SSS). Both juvenile and adult growth are also likely to be linked with large scale climatic indices such as the Niño-4 index that affects SST along the shelf of Western Australia^13^,^14^ and the Pacific Decadal Oscillation (PDO), which is associated with precipitation^15^ (see Methods section for further details).

We used a dendrochronology approach to generate records of growth for both the juvenile and adult stages of mangrove jack collected from north-western (NW) Australia. These records were obtained from otoliths (fish ear stones), which contain annual growth increments. Otolith growth and somatic growth are tightly linked, thus, time series of annual growth can be assembled using otolith increment measurements^16^,^17^. We generated a master growth chronology based on records from a sample of individuals and tested for relationships with the physical oceanographic and atmospheric variables outlined above. These analyses enabled us to identify important drivers of growth for each life history stage. Our results were then evaluated with respect to the susceptibility of this species to predicted patterns of climate change in both adult and juvenile habitats.

## Results

### Chronology development for adults and juveniles

The chronology derived from the 36 adult fish covered the period from 1975 to 2003, a 29-year series (see Supplementary Fig. S1 online). Measurements from at least 20 fish contributed to each year value, with more than 28 fish contributing to the period between 1979-2002 and all fish (n = 36) contributing to the years 1988-1995. Even though the common variance among individuals was low (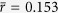 with s.e. = 0.016), the sample depth was sufficient to show statistically significant, positive 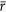 for all periods from 1975 to 2003, suggesting that there were clear, synchronous growth patterns for these fish in that period. The averaged expressed population signal (EPS) for the same period was 0.84, indicating that the adult chronology well-represented the theoretical population chronology. In most of our samples, the signature years of 1989-1990 had conspicuously wide increments, while the years 1991-1992 had conspicuously narrow increments. As the juvenile chronology only included increment width data from age 1 to 9, the series were too short to obtain robust 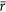 values, however a continuous 14 year series (1965-1978; see Supplementary Fig. S1 online) with a sample depth of at least 10 fish contributing to each year was selected for analysis with environmental parameters.

### Relationships between chronologies and environmental drivers

Correlations were calculated between 15 variables (January, February and March values for each of the 5 environmental factors) and both the adult and juvenile chronologies across the relevant time periods. Following the approach of Black *et al*.^18^ we reduced the level of significance from p < 0.05 to p < 0.03 to account for multiple comparisons.

**Figure 1:**
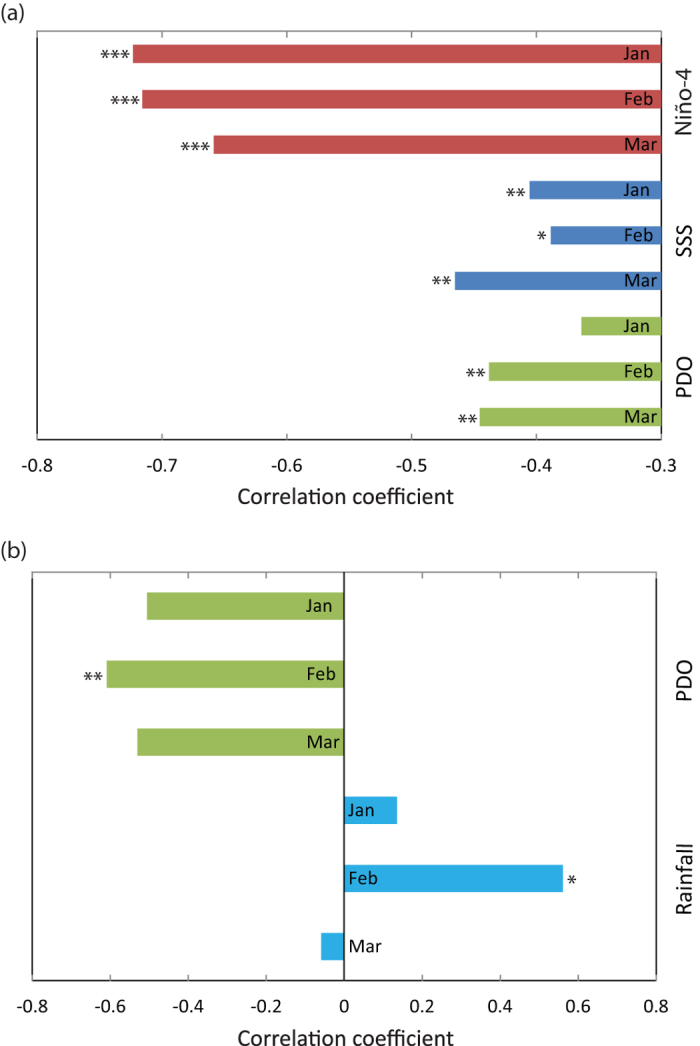
Pearson’s correlation coefficients between *Lutjanus argentimaculatus* chronologies and January to March values of significantly correlated environmental variables: (**a**) adult chronology from 1975 to 2003 with the Niño-4 index^39^ (Niño-4), Sea Surface Salinity^41^ (SSS) and Pacific Decadal Oscillation^40^ (PDO) and (**b**) juvenile chronology from 1965 to 1978 with PDO and rainfall. Asterisks represent p-values of the Pearson’s correlation test, with p < 0.01 (***), p < 0.03 (**) and p < 0.04 (*).

**Figure 2:**
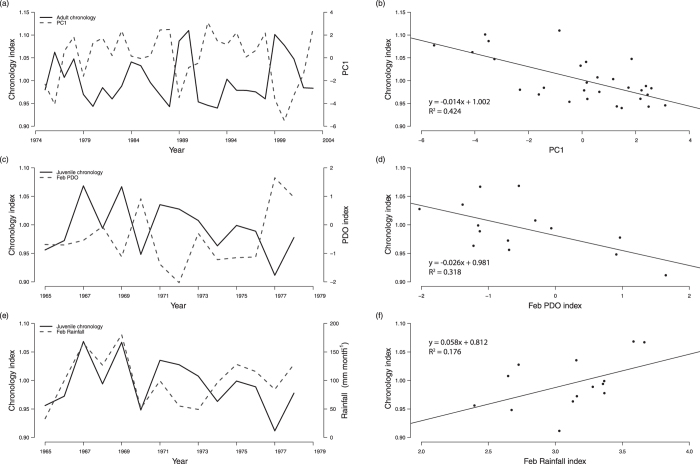
Relationships between *Lutjanus argentimaculatus* chronologies and environmental variables included in the linear models: (**a**) adult chronology from 1975 to 2003 plotted with Principal Component 1 (PC1), which accounted for 60% of the variation for January to March values of the Niño-4 index^39^ (Niño-4), Sea Surface Salinity^41^ (SSS) and Pacific Decadal Oscillation^40^ (PDO); (**b**) regression plot of adult chronology with PC1; (**c**) juvenile chronology from 1965 to 1978 with February PDO index^40^; (**d**) regression plot of juvenile chronology with February PDO index^40^; (**e**) juvenile chronology from 1965 to 1978 with February rainfall (mm month^-1^); and (**f**) regression plot of juvenile chronology with fourth root transformed February rainfall values.

**Figure 3:**
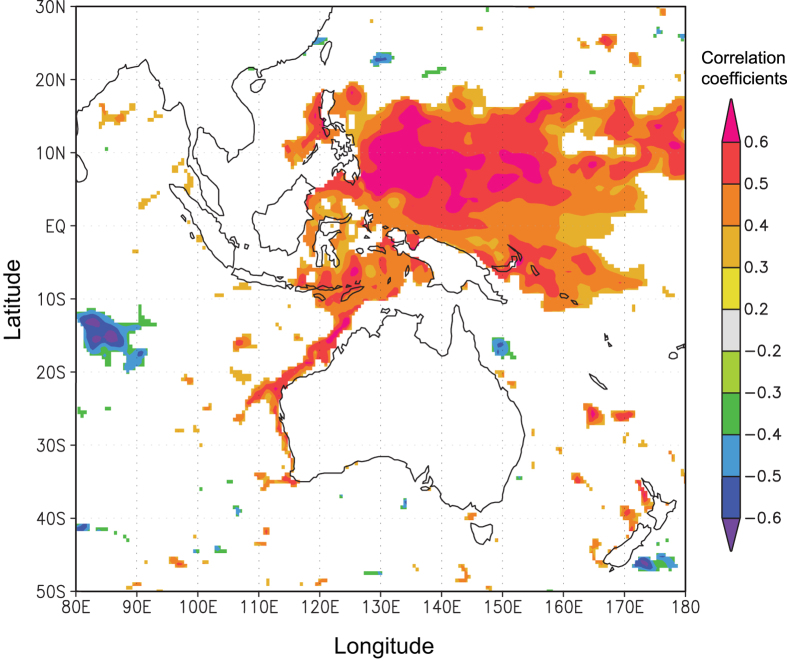
Map of spatial correlations of adult *Lutjanus argentimaculatus* chronology with ocean heat content. January ocean heat content (0–750 m depth) from Simple Ocean Data Assimilation (SODA) reanalysis of ocean climate variability^42^ was correlated with the adult mangrove jack chronology from 1975 to 2003. Warmer colours indicate positive correlations, cooler colours indicate negative correlations. Map was obtained and modified from KNMI Climate Explorer^43^, a web application for climate data, using our data and ocean heat content.

The growth chronology of adult mangrove jack over the period 1975-2003 was negatively correlated with the Niño-4 index, SSS and PDO (Fig. 1a) and positively correlated with rainfall in March (see Supplementary Table S2 online for results of all comparisons with environmental variables). Because of collinearity among the Niño-4 index, SSS and PDO, these variables were combined in a principal components analysis. The first principal component (PC1) explained 60% of the total variation and the second (PC2) 18%. All variables had similar positive loadings on PC1 (range 0.310 - 0.357) and there was a negative correlation between the adult chronology and PC1 (Fig. 2a). A linear regression model that related PC1 to the adult chronology was significant (p = 0.00008) with an adjusted R^2^ = 0.424 (Fig. 2b). As there was a significant correlation between the adult chronology and rainfall in March (see Supplementary Table S2 online), this environmental variable was fourth root transformed (due to the range of data) and included in the linear model. However, the resulting model was not significant (p = 0.391). Strong positive correlations between the adult chronology and ocean heat content were identified in the map of spatial correlations, notably in the areas of the Pacific Warm Pool in the western Pacific, in eastern Indonesian Seas and along the coast of NW Australia (Fig. 3).

The juvenile chronology was significantly (p < 0.03) and negatively correlated with PDO in February (Figs. 1b,2c,d) and there was a weaker positive correlation with rainfall in the same month that was marginally non-significant (p = 0.037; Figs. 1b,2e,f).These two environmental variables were not collinear (r = −0.096, p = 0.745), so both were included in a linear model with the juvenile chronology. Although rainfall in February was marginally non-significant in the correlation analyses (Fig. 1b), the addition of this variable greatly improved the strength of the significant (p = 0.010) linear model (adjusted R^2^ = 0.490 compared to R^2^ = 0.318 without rainfall in February; Fig. 2d).

## Discussion

Our study is the first to develop growth chronologies from the otoliths of both adult and juvenile stages of a tropical coastal fish. Although fractional common variance (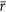) was low for the adult chronology, we found significant environmental drivers of otolith growth. The quality of the juvenile chronology could not be assessed using 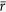 values due to the relatively short length of each individual series (1-9 years), but our results show that the growth patterns of both adult and juvenile (albeit with a lesser amount of certainty) mangrove jack show significant responses to environmental variables.

The strongest correlation between the growth chronology of adults and environmental signals involved a large-scale (1000s of km) SST variable (Niño-4 index). The Niño-4 index is defined as the westernmost region between 5°N-5°S and 160°E-150°W where the El Niño-Southern Oscillation (ENSO) variations lead to significant SST changes. This implied that events occurring in the western Pacific affected adult growth, presumably via the Indonesian Through-Flow (ITF), an oceanic connection between the western Pacific and the Indian Ocean through current flow from the Indonesian Seas. The strength of the ITF is greater during La Niña conditions (in our datasets, notably during 1988-1989 and 1999-2000), when warmer and lower salinity waters are transported to the NW coast of Australia. This is thought to account for higher SST on the NW coast during La Niña years compared to El Niño years^14^,^19^. When La Niña events are exceptionally strong, this may result in anomalously warm waters along the NW coast, as was the case during the summer of 2010/2011^13^. For mangrove jack, the growth chronology suggested that conditions of warm and low salinity waters during strong La Niña years were correlated with higher growth rates. Despite this pattern we did not find any correlation of growth with SST at regional (100s of km) scales, a result that contrasts with other studies that have recorded positive correlations of growth chronologies with regional SST in temperate species such as flatfish (*Limanda aspera*)^17^, red and grey snapper (*Lutjanus campechanus* and *L. griseus*)^18^, luderick (*Girella tricuspidata*)^20^ and western blue groper (*Achoerodus gouldii*)^7^. However, we did find strong correlations with ocean heat content, which implies that a combination of both temperature and salinity affects growth more strongly than either individual variable.

As fish are poikilotherms, it might be expected that they should grow faster when water temperatures are warmer, assuming that they are not at thermal limits. Aside from warmer temperatures during La Niña events, the decreased salinity may also influence growth through osmoregulation or food conversion efficiency^21^. Many studies have found higher growth rates of fish correlated with a lower standard metabolic rate, which occurs at intermediate salinities below that of seawater (see review by Boeuf & Payan^21^). Similarly, the lower salinity waters associated with La Niña events might also lower metabolic costs and allow more energy to be allocated to growth of adults in coastal waters. A study of Atlantic cod (*Gadus morhua*) found higher growth rates at lower salinities, resulting from increased food conversion efficiency^22^. This may be another possible mechanism influencing growth of mangrove jack in NW Australia, provided that the productivity during La Niña events is also high (and thus the species is not food-limited). In any event, our results suggest that the interaction of both temperature and salinity are likely to be affecting adult growth.

The growth of juvenile mangrove jack was influenced by environmental variables that were linked both directly and indirectly (via the PDO) to rainfall patterns. Positive correlations with rainfall were expected because lower salinities are likely to increase growth rates (as noted above). A negative correlation with the PDO could reflect the influence of this variable on rainfall, via SST anomalies in the Pacific that in turn generate precipitation and convection heating anomalies^23^. In eastern Australia negative PDO values generate cool and wet conditions^24^. However, we found that the PDO and rainfall were not correlated in the NW region over the juvenile time period. It may be possible that the PDO affects some other, unknown local factor that was not tested. Irrespective of the link between growth and the PDO, the effects of rainfall on the estuarine phase of the species are more obvious. Increases in rainfall lead to greater river runoff and subsequent declines in salinity in estuaries, a process that will be exacerbated by the small size and shallow depths of these habitats on the NW coast^6^. Previous studies on juvenile grey snapper (*L. griseus*) have found that growth efficiencies decrease at higher salinities^25^ and laboratory experiments have shown that they prefer lower salinities that minimize osmoregulatory costs^26^. Juvenile turbot (*Scophthalmus maximus*) have also shown better food conversion efficiencies at lower salinity levels^27^, so juveniles of mangrove jack might respond in similar ways to changes in salinity from increased rainfall. Additionally, there might be indirect effects of salinity in estuarine habitats such as salinity-induced changes to prey, predator or competitor abundances^26^.

While mindful of the caveat that the adult and juvenile chronologies do not represent the same time period, we show that the physical and biological phenomena that drive growth patterns of the mangrove jack are likely to differ between juvenile and adult life history stages. This result implies that the responses of the individuals to changing climate conditions may vary with ontogeny. Rainfall in NW Australia has been increasing from the 1970s to the present day and predicted increases in mean precipitation during the Asian-Australian monsoon coupled with an intensification of ENSO-induced rainfall variability^28^ might lead to better growth rates for juveniles. However, the pattern of Indo-Pacific warming due to climate change is also predicted to result in a greater frequency of El Niño-like conditions which might reduce the strength of the ITF^29^, and could negatively affect the growth of adult mangrove jack. These contrasting effects complicate predictions of the response of this species to climate change in the future. As the patterns of ontogenetic movement of mangrove jack are common to many species, our study suggests that it may be very difficult to generalise on the likely outcomes of climate change for a large suite of fishes in tropical coastal environments.

Because of the ubiquity of ontogenetic changes in habitat by fishes in nearly all marine environments, our results have implications for many species. The degree of complexity of the effect of climate change on a species may be dependent on the scale, degree and timing of change in habitats between life history stages, and the impacts could be reduced for those species that show only limited patterns of movement. However, even those species that do not change habitat are likely to change diet and trophic role with age. Although the biochronology approach addresses only population growth anomalies inferred from otoliths, these studies are now required to determine if trophic changes add a similar layer of complexity to the prediction of the effects of climate change as those of ontogenetic movement. In addition, the climate-growth relationships obtained from chronology-based studies will be a critical component of ecosystem-based fishery management.

## Methods

### Study species

The mangrove jack (*L. argentimaculatus*) is long-lived, with some individuals exceeding 50 years of age^30^. It is a prized target of commercial, artisanal and recreational fisheries throughout its range, which includes much of the tropical Indian Ocean and western Pacific. In Western Australia, estimated recreational catches of this species are approximately 2.7 tonnes^31^ and commercial catches are approximately 13 tonnes in 2011-12^32^. Individuals exhibit negative exponential growth rates early in ontogeny, with lengths of around 400 and 550 mm attained in 5 and 10 years respectively^30^, and an observed maximum length of around 800 mm from the sample collection. Juveniles have been shown to inhabit estuaries and mangroves, before moving offshore at around 7 years old^30^.

### Study site

**Figure 4:**
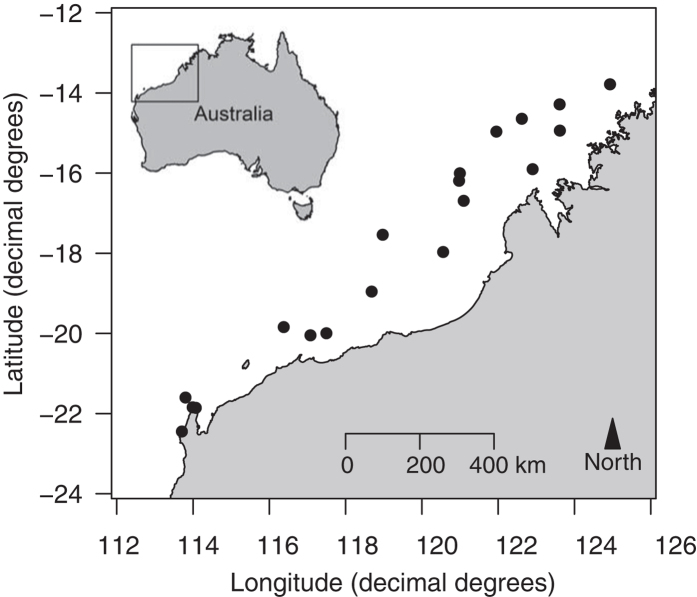
Map of sampling sites in north-western Australia. *Lutjanus argentimaculatus* were captured from various offshore sampling areas by research trapping, commercial trapping or commercial fish trawls from 1996 to 2005. Points on the map show general locations of capture and the map was created using the package “mapdata”^44^ in R^38^ software.

The tropical NW coast of Australia is characterised by a large tidal range, a high frequency of cyclones, and warm, oligotrophic surface waters. These low salinity waters largely emanate from the Indonesian Through-Flow (ITF), which connects waters in the western Pacific to the Indian Ocean^29^. The NW coast consists of numerous small barrier and fringing reefs in shallow water^33^. In this region, the maximum rainfall occurs in summer, coinciding with the highest rate of tropical cyclone occurrence. About 30 river basins drain into the NW coast, however, the majority of the river flows enter the coastal waters north of ~18°S^33^ (Fig. 4). River flow in the north-west has been reported by Lough^33^ to be more variable compared to river flow on the east coast and across Australian rivers in general. Estuaries on the NW coast are small and shallow compared to global standards^6^, so are likely to have variable environments heavily influenced by terrestrial inputs such as river flows.

### Sample collection

Archived otolith collections of *L. argentimaculatus* were obtained from the Department of Fisheries of Western Australia. These had been collected from the Pilbara and Kimberley regions of NW Australia from commercial catches (fish trawls and fish traps) and recreational line catches, supplemented with research sampling using fish traps between 1996 and 2005 (see Pember *et al*.^30^ for details). Adult fish were caught from offshore reefs across the continental shelf of NW Australia (Fig. 4). The sagittal otoliths of each fish were removed, cleaned and stored to dry in paper envelopes. One sagittal otolith from each fish was embedded in epoxy resin and sectioned transversely through the primordium in a direction perpendicular to the sulcus acusticus, using a low speed saw with a diamond tipped blade (Buehler, United States of America, USA). Sagittal otolith sections were cut into two or three 150-300 μm thick sections near the core of the otolith to enhance interpretation. These sections were cleaned and mounted on glass slides using DePeX mounting medium^30^.

The formation of annual increments in the otoliths of the mangrove jack has been validated using marginal increment analysis by a number of studies^30^,^34^. Each increment within the otolith consists of translucent and adjacent opaque zones with the latter completed between October and November each year^30^. The spawning season takes place between September and December each year^30^, and growth is assumed to occur during the warm summer months of January to March for both juveniles and adults. Only otoliths from fish at least 23 years old (to ensure sufficient years for chronology development) with clear increments (to increase accuracy) were selected for image analysis (n = 36), with 3 fish discarded due to poor clarity. The selection of otoliths with clear increments may lead to a possibility of sample bias, however the importance of being able to correctly identify each increment and accurately assign calendar years was essential to the study. Selected fish were collected over the years 1996-2005, with most collected in 2003 and 2004. The ages of fish ranged from 24-52 years with an average of 37 years. The samples had a standard length range of 428-700 mm with an average of 558 mm.

### Image analysis

**Figure 5:**
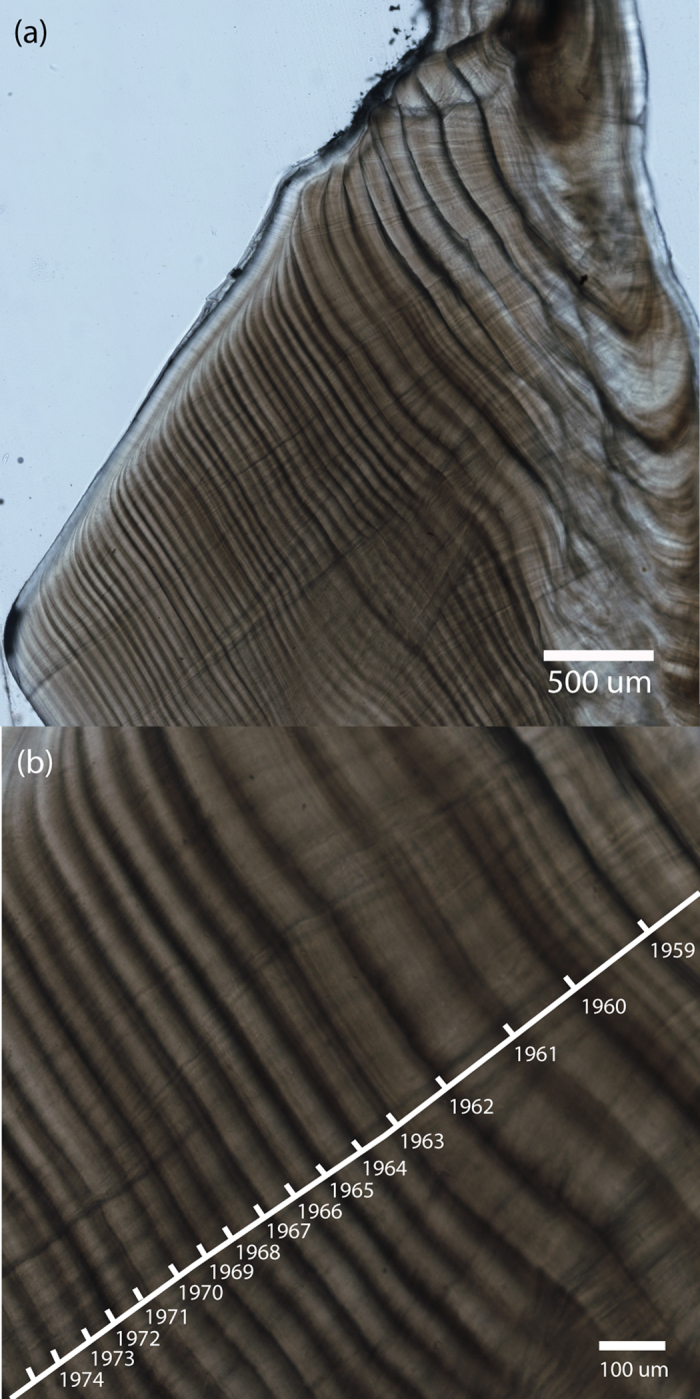
Photomicrographs of the dorsal side of a *Lutjanus argentimaculatus* otolith section: (**a**) the dorsal section of the otolith was chosen for consistently clear annual increments and (**b**) a close up image of the same otolith with transect line and increments labelled with corresponding calendar years.

Multiple sections of the same otolith were examined under a dissecting microscope to assess the clarity of growth increments and the best section was used for measurements. The region next to the sulcus on the dorsal side of each otolith was imaged using an Olympus IX81 inverted microscope (Olympus Corporation, Tokyo, Japan) with a ProScan II motorised stage (Prior Scientific, Japan). Multiple images were captured in brightfield (transmitted light) using a Nikon Digital Sight DS-2Mv colour camera (Nikon, Japan) with a 10× objective (Olympus UPlan 10 × 0.3 NA dry objective) and stitched using NIS-Elements AR software (version 3.22 & 4.13, Nikon, Japan). This region of the otolith was chosen because it was consistently the clearest region for annual increments (Fig. 5a). Increment widths were measured using a plugin (“IncMeas”)^35^ written for the image analysis software, Image J (version 1.48, National Institutes of Health, USA). Three transects parallel to the direction of growth were drawn on each image montage. The outer edge of the opaque zone of each increment was marked along each transect (Fig. 5b). Increment widths were measured and recorded along with the inferred calendar year of formation, obtained by working backwards from the year of capture and taking into consideration the approximate timing of completion of each increment. This was part of the visual crossdating process, which assumes that the environment induces synchronous, time-specific growth patterns that can be matched among individuals. Beginning with the clearest otolith sections in which all increments were easily identified, conspicuously narrow or wide increments were noted (referred to as signature years that should be synchronous among samples) and used to ensure the correct assignment of calendar years to increments^36^.

### Otolith chronology development

We used the program COFECHA, from the International Tree-Ring Data Bank Program Library to assist with statistical crossdating^36^. Otolith increment series from three transects per fish (obtained from Image J measurements) were loaded in COFECHA and detrended to remove ontogenetic trends while retaining high frequency variation. A spline rigidity parameter of 22 years was used, following the methods of Black *et al*.^36^. Measurements were standardized by dividing by the spline fit, and then the correlation between each standardized series and the average of all other standardized series was calculated to allow assessment of series alignment. Samples with low correlations were visually inspected for potential errors in increment boundary placements (e.g., missed marking a year due to a faint opaque zone), and any obvious errors were corrected.

For chronology development, we followed Black *et al*.^17^ in using a method similar to regional curve standardization, which is used in tree-ring research to remove ontogenetic trends while preserving low-frequency variations in ring width. Increment widths were aligned by fish age and the mean increment width at each age was calculated. Each series was then divided by the mean-by-age series to obtain standardized values. Multiple standardized series belonging to the same fish were averaged before inclusion in the chronology.

Standardized measurements were classified as either “adult” or “juvenile” years and two chronologies were created to allow the detection of different drivers of growth in the two phases. The adult chronology was constructed with all 36 fish using increments formed after age 7 (based on an estimated age of 7 years for sexual maturity for >50% of mangrove jacks caught in Western Australia^30^). Only years in the chronology with a sample size of at least 20 fish were retained so that variation would be more likely to reflect synchronous growth patterns among individuals, rather than being strongly affected by variation in growth of only one or two fish. Standardized series of increment width measurements of adults from the selected years were averaged to create a single chronology representative of adult fish. The juvenile chronology was constructed using data from age 1 to 9 since >90% of mangrove jacks caught in Western Australia are mature by 9 years of age^30^. Additionally, this produced more series overlap and a longer chronology than if 7 years had been used. A minimum sample size of 20 fish was not possible for the juvenile dataset, hence the longest continuous sequence of years in which sample depth exceeded 10 fish was selected, resulting in a sample size of 30 fish.

The quality of the chronology was assessed using the mean of pairwise series correlations (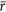), an estimate of fractional common variance, as well as expressed population signal (a measure of how well the chronology represented the theoretical population chronology) using the package “dplR”^37^ in R^38^ software (version 3.0.1).

### Correlations with environmental parameters

Pearson’s correlations and the linear models relating otolith chronologies to a number of environmental factors were calculated using R^38^ software. Assumptions of normality, homoscedasticity and independence were verified for all linear models and Durbin-Watson tests were used to check for any autocorrelation of residuals. Five environmental factors were used in the analysis, with only January to March values retained to coincide with the growing season for fish. Hence, a total of 15 variables were considered, 3 months (January, February and March) for each of the 5 factors. These 5 environmental factors were large-scale climate indices such as the Niño-4 index^39^ (based on sea surface temperature anomalies over the central Pacific using HadISST1), Pacific Decadal Oscillation index^40^ (PDO; based on sea surface temperature anomalies over the North Pacific using HadSST3), as well as regional environmental variables including Sea Surface Temperature^39^ (SST), Sea Surface Salinity^41^ (SSS) and rainfall (Bureau of Meteorology, Australian Government). Principal Components Analysis (PCA) was used to deal with collinearity between the Niño-4 index, SSS and PDO over the period 1975-2003. Nine environmental variables (January, February and March values for each of the 3 collinear factors) were put into the PCA. Ocean heat content (incorporating temperature and salinity from 0–750 m depth) from the Simple Ocean Data Assimilation (SODA) reanalysis of ocean climate variability^42^ was used to examine the interactions of temperature and salinity on the growth of adults. All data were obtained from the Royal Netherlands Meteorological Institute (KNMI) Climate Explorer^43^, a web application for climate data (http://climexp.knmi.nl), unless otherwise stated. Regional data were obtained from an area covering the latitudes 23°S to 14°S and longitudes 113°E to 127°E, which includes the Pilbara and Kimberley regions of NW Australia (Fig. 4).

## Additional Information

**How to cite this article**: Ong, J. J. L. *et al*. Contrasting environmental drivers of adult and juvenile growth in a marine fish: implications for the effects of climate change. *Sci. Rep*. **5**, 10859; doi: 10.1038/srep10859 (2015).

## Supplementary Material

Supplementary Information
