# Supplementary material for: Contrasting environmental drivers of adult and juvenile growth in a marine fish: implications for the effects of climate change
Source: Sci Rep. 2015 Jun 8;5:10859. doi: 10.1038/srep10859 (PMC4460954; doi:10.1038/srep10859)
Supplement: Supplementary Information [file srep10859-s1.pdf]

Supplementary Information

Title: Contrasting environmental drivers of adult and juvenile growth in a marine fish: implications for the effects of climate change

Authors: Joyce Jia Lin Ong[1,2,*], Adam Nicholas Rountrey[3] , Jessica Jane Meeuwig[1], Stephen John Newman[4], Jens Zinke[2,5,6,7] and Mark Gregory Meekan[2]

Institute:

[1] School of Animal Biology and the Centre for Marine Futures (UWA Oceans Institute M096), University of Western Australia, 35 Stirling Highway, Crawley, Western Australia 6009

[2] Australian Institute of Marine Science, UWA Oceans Institute (M096), University of Western Australia, 35 Stirling Highway, Crawley, Western Australia 6009

[3] Museum of Paleontology, University of Michigan, 1109 Geddes Avenue, Ann Arbor, Michigan 48109-1079, United States of America

[4] Western Australian Fisheries and Marine Research Laboratories, Department of Fisheries, Government of Western Australia, PO Box 20, North Beach, Western Australia 6920

[5] School of Earth and Environment and the UWA Oceans Institute (M096), University of Western Australia, 35 Stirling Highway, Crawley, Western Australia 6009

[6] School of Geography, Archaeology and Environmental Studies, University of Witwatersrand, Johannesburg, South Africa

[7] Curtin University of Technology, Department of Environment and Agriculture, Kent Street, Perth, Western Australia 6845

Supplementary Figure S1: Raw, undetrended and detrended increment widths of all fish used, supporting the adequacy and confidence of the final chronologies used.

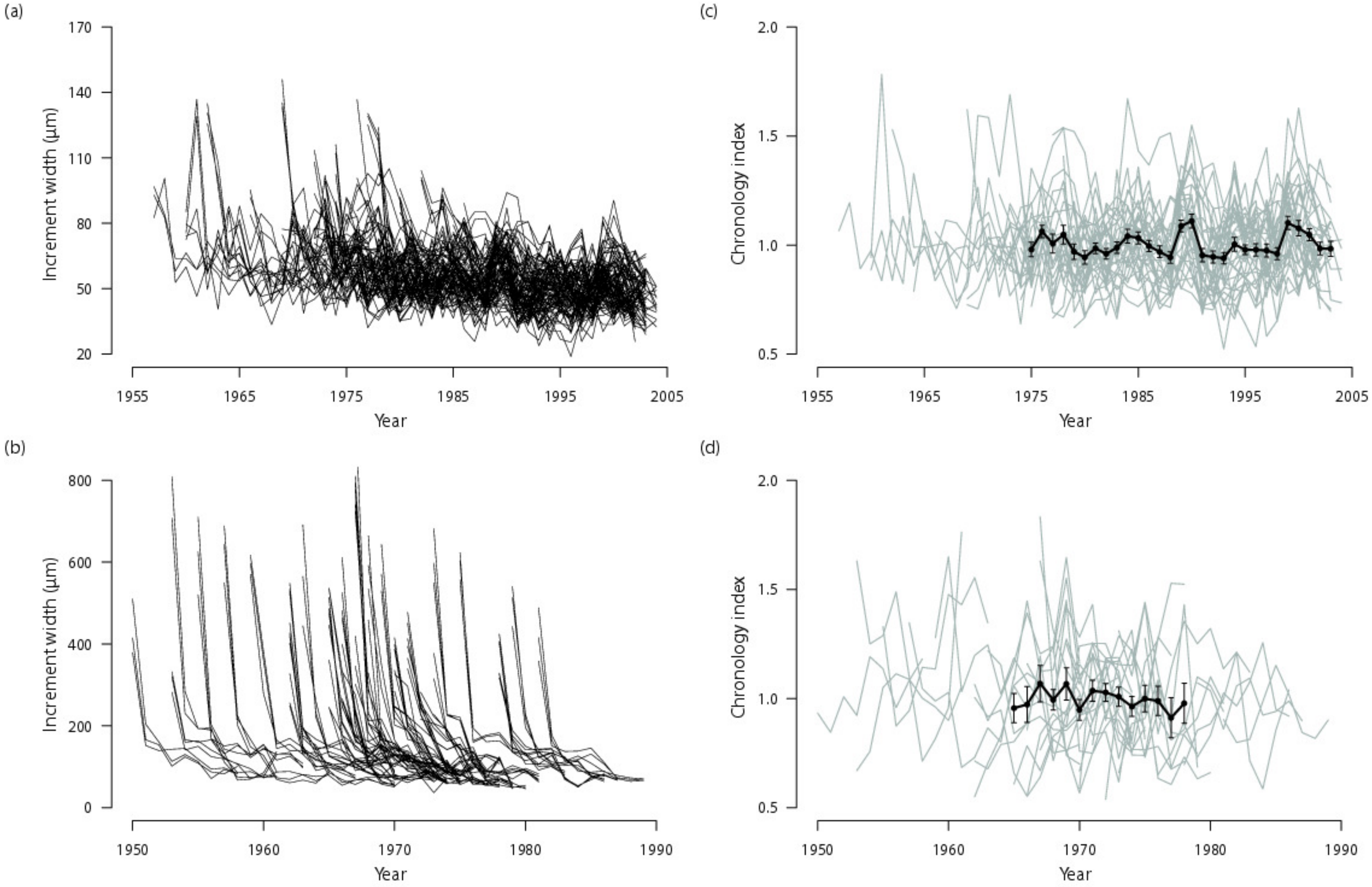

Supplementary Figure S1. Raw increment width time series, detrended and final chronologies used from the otoliths of *Lutjanus argentimaculatus*: raw increment widths of adults (a) and juveniles (b), detrended and final chronologies with standard error bars of adults (c) and juveniles (d).

Supplementary Table S2: Full list of Pearson's correlation coefficients and respective p-values for adult and juvenile chronologies tested against the environmental variables.

Supplementary Table S2. Pearson's correlation coefficients (R) and p-values (P) of adult and juvenile *Lutjanus argentimaculatus* with all 15 environmental variables. The adult chronology and relevant environmental values were from 1975-2003 while the juvenile chronology and relevant environmental values were from 1965-1978. A reduced level of significance ($p < 0.03$) was used to account for multiple comparisons. Niño-4 = Niño-4 index, SSS = Sea Surface Salinity, PDO = Pacific Decadal Oscillation, SST = Sea Surface Temperature.

Life stage	Variable	Month	R	P
Adult	Niño-4	Jan	-0.723	0.000009
Adult	Niño-4	Feb	-0.716	0.00001
Adult	Niño-4	Mar	-0.659	0.0001
Adult	SSS	Jan	-0.406	0.029
Adult	SSS	Feb	-0.389	0.037
Adult	SSS	Mar	-0.465	0.011
Adult	PDO	Jan	-0.364	0.052
Adult	PDO	Feb	-0.438	0.017
Adult	PDO	Mar	-0.445	0.016
Adult	Rainfall	Jan	0.072	0.710
Adult	Rainfall	Feb	-0.106	0.585
Adult	Rainfall	Mar	0.435	0.018
Adult	SST	Jan	0.201	0.296
Adult	SST	Feb	0.245	0.200
Adult	SST	Mar	0.114	0.557
Juvenile	Niño-4	Jan	0.000	0.999
Juvenile	Niño-4	Feb	0.034	0.908
Juvenile	Niño-4	Mar	-0.026	0.930
Juvenile	SSS	Jan	0.072	0.807
Juvenile	SSS	Feb	0.174	0.552
Juvenile	SSS	Mar	0.103	0.727
Juvenile	PDO	Jan	-0.506	0.065
Juvenile	PDO	Feb	-0.609	0.021
Juvenile	PDO	Mar	-0.530	0.051
Juvenile	Rainfall	Jan	0.135	0.645
Juvenile	Rainfall	Feb	0.560	0.037
Juvenile	Rainfall	Mar	-0.059	0.841
Juvenile	SST	Jan	0.425	0.129
Juvenile	SST	Feb	0.457	0.101
Juvenile	SST	Mar	0.298	0.301
